# The effects of mothers’ musical background on sedentary behavior, physical activity, and exercise adherence in their 5-6-years-old children using movement-to-music video program

**DOI:** 10.1371/journal.pone.0195837

**Published:** 2018-04-18

**Authors:** Pipsa P. A. Tuominen, Jani Raitanen, Pauliina Husu, Urho M. Kujala, Riitta M. Luoto

**Affiliations:** 1 The UKK Institute for Health Promotion Research, Tampere, Finland; 2 Faculty of Sport and Health Sciences, University of Jyväskylä, Jyväskylä, Finland; 3 Faculty of Social Sciences, Health Sciences, University of Tampere, Tampere, Finland; University of Zurich, SWITZERLAND

## Abstract

**Objectives:**

The purpose of this study was to examine whether mothers’ musical background has an effect on their own and their children’s sedentary behavior (SB) and physical activity (PA). The aim was also to assess children’s and their mothers’ exercise adherence when using movement-to-music video program.

**Design:**

Sub-group analysis of an intervention group in a randomized controlled trial (ISRCTN33885819).

**Method:**

Seventy-one mother-child-pairs were divided into two categories based on mothers’ musical background. Each pair performed 8 weeks exercise intervention using movement-to-music video program. SB and PA were assessed objectively by accelerometer, and exercise activity, fidelity, and enjoyment were assessed via exercise diaries and questionnaires. Logistic regression model was used to analyze associations in the main outcomes between the groups.

**Results:**

Those children whose mothers had musical background (MB) had greater probability to increase their light PA during the intervention, but not moderate-to-vigorous PA compared to those children whose mothers did not have musical background (NMB). SB increased in both groups. Mothers in the NMB group had greater probability to increase their light and moderate-to-vigorous PA and decrease their SB than mothers in the MB group. However, exercise adherence decreased considerably in all groups. Completeness, fidelity, and enjoyment were higher among the NMB group compared to the MB group.

**Conclusions:**

The present results showed that mothers without musical background were more interested in movement-to-music exercises, as well as their children. For further studies it would be important to evaluate an effect of children’s own music-based activities on their SB and PA.

## Introduction

The current physical activity (PA) guidelines for children recommend at least 180 minutes activity at any intensity spread throughout the day [1,2]. Furthermore, excessive sitting should be avoided [2,3]. Adults are recommended to engage in regular moderate intensity physical activity for at least 150 minutes per week, vigorous PA for minimum of 75 minutes per week or a combination of these (moderate-to-vigorous PA, MVPA). Strength training should be performed at least twice a week and time spent in sedentary behaviors (SB) should be minimized [3,4].

In everyday context, recent studies have measured daily amount of PA objectively during one week by a hip-worn accelerometer [5,6] which detects overall PA and SB validly and reliably [7,8]. In laboratory context, energy efficiency and work output are widely measured and examined in relation to the psychological and physiological factors [9–12]. Questionnaires before and/or after exercise session or longer PA program are widely used to assess the psychological aspects of PA performance [5,13]. In addition, for assessing engagement or adherence to a physical training program in everyday context, diaries and/or questionnaires are needed to find out the frequency of exercises and length of a single exercise session [6,13].

The most common music activities are listening, singing, playing an instrument, and exercising, moving or dancing to music. Musical background can be defined as an engagement of activity, which means the individual’s active involvement or participation in the music-based activity, or formal music training [14,15]. Assessing musical behavior instead of musicality, musical abilities, or skills is relatively recent approach to assess musical background [14,16]. Studies documenting use of the music in children have found that 5–6-years-olds respond favorably towards involvement in all musical activities, but they prefer moving and playing based activities [17,18]. Movement-to-music is often included to PA programs for children, and regularly provided, structured exercises have been found to increase the amount and intensity of PA, as well as improve their motor skills [6,19]. Among adults, studies documenting the benefits of the music in sport and exercise context have found that music could increase exercise adherence and participation [9], and motivational music has found enhanced affect, reduced ratings of perceived exertion, improved energy efficiency, and lead to increased work output [10–12].

Completeness and fidelity have been used in earlier studies to explain the success of implementation, as well as evaluation tool for conceptual model to support increasing PA in children [20]. Based on a conceptual framework by Carroll et al. (2007) completeness is defined as quantity or perfection of dose, i.e., how fully the intervention components are met or whether all the people who should be participating in actually do so [21,22]. Fidelity is defined as quality of the intervention components, i.e., how well the intervention components are met or whether all the people implement the content of program [21,22].

Enjoyment of the activity may be an important element for exercise adherence and motivation. Remmers et al. (2015) found that enjoyment of PA was related with active behavior, specifically all PA intensities combined, in children [5]. Among adults, it was suggested that activities they like will be continued with greater engagement and adherence compared to activities they don’t like [13,23]. It is also known that individuals with musical background, specifically with formal music training, use music less for entertainment than individuals without musical background in their everyday life [15]. This might be critical in music-based exercises, because the long-term health benefits require regular engagement in PA and reduction of SB.

Due to lack of previous studies in the combined field of movement-to-music exercises in the home environment and musical background, our aim is to study whether mothers’ musical background (i.e., music-based hobbies or profession) have an effect on their own and their children’s SB and PA. Based on earlier studies we are interested whether mothers and children without musical background could decrease SB and increase PA more than mothers and children with musical background. We also study children’s and their mothers exercise adherence when using movement-to-music video program.

## Materials and methods

### Participants and study design

The current study is a subgroup analysis of an intervention group in randomized controlled trial (RCT) called Moving Sound [24,25], in where the main outcomes were children’s and their mothers’ sedentary time and PA during eight weeks’ period. The participants of the Moving Sound study were recruited between November 2014 and January 2016 from the cohort of NELLI: Pregnancy as a window to the future health of mothers and children, the 7-year follow-up of a gestational lifestyle intervention in the Pirkanmaa area, Finland (ISRCTN33885819; http://www.controlled-trials.com/).

The Moving Sound study was approved by the Pirkanmaa Ethics Committee in Human Sciences (Tampere, Finland), and RCT was registered at ClinicalTrials.gov (NCT02270138). All the mothers provided written consent on their and their child’s behalf.

In this study, only those mother-child pairs who belonged to the Moving Sound intervention group and answered to the questions about mothers’ musical background (71 mother-child pairs) were included to the analyses. For the analyses, the intervention group was divided into two categories based on mothers’ musical background. The musical background was defined as musical behavior including four items: playing an instrument, singing, listening to music, and dancing or having other movement-to-music activities. In order to study the participants’ musical background similar to previous studies [15], we asked their formal or informal music training and movement-to-music activities, the number of years of these music-based activities, and their activity as music listeners. To indicate mothers’ musical background, they had to had music as their job, were studying music professionally, or three out of four items listed above had to be positive (music as a hobby).

All mothers and children were instructed to use an accelerometer every day during waking hours for weeks one (baseline/reference week), two (the first intervention week), and eight (the last intervention week). Further, for the same weeks mothers were instructed to complete exercise diaries (the type and duration of exercise) for themselves and their child. Mothers and children were instructed to use the movement-to-music video program DVD every other day from the beginning of week two to the end of week eight. The movement-to-music video program was based on PA recommendations and included three separate exercise programs, each lasting 10 minutes. In order to allow mother and child to choose the suitable amount of exercise for themselves, the videos could be used individually or consecutively. In addition, every movement had one to three variations. The detailed contents of video program have been previously described by Tuominen et al. (2015).

### Measurements

The primary outcomes of the study were SB and PA, which were assessed objectively by the triaxial hip-worn accelerometer (Hookie AM20, Traxmeet Ltd, Espoo, Finland) for three weeks (baseline week, the first intervention week, and the last intervention week). Participants were instructed to use the accelerometer in elastic belt on their right side of the hip during waking hours for 7 consecutive days on each measurement week, excluding water-based activities (e.g., shower or swimming). The data was analyzed as the mean amplitude deviation (MAD) of the resultant acceleration for each 6-second epoch [7]. The MAD values were further converted to metabolic equivalents (MET) and intensity was calculated of these estimated MET values [7]. Lying and sitting down (< 1.5 MET) were combined to SB, standing still (< 1.5 MET) and light PA (LPA 1.5–2.9 MET) were analyzed separately, and further, moderate PA (MPA 3.0–5.9 MET) and vigorous PA (VPA ≥ 6.0 MET) were combined as moderate-to-vigorous PA (MVPA) [26,27].

The secondary outcome of the study was exercise adherence which was divided for exercise activity with movement-to-music video (completeness), fidelity (quality), and enjoyment examined via the exercise diaries and questionnaires. Mothers were also asked to assess the motivational effects of songs in the video using Brunel Music Rating Inventory (BMRI-2) [28,29]. Based on these assessments mothers were classified to one of the three groups: highly, moderately, or neutrally motivated by music. Exercise diaries were completed during the same weeks when accelerometer was used. Mothers were asked to fulfill the diaries for themselves and for their child’s behalf. Questionnaires were completed at baseline, at the end of the first intervention week, and at the end of the study.

Completeness was defined as adherence to training program based on number of completed exercises with movement-to-music video program during the first and the last intervention week. Self-reported number of exercises were based on diaries. Fidelity was defined as the content of the exercise, and it was assessed via questionnaires to find out whether the children and their mothers moved as instructed during the video watching. Enjoyment was defined as children and their mothers having fun with the video program. The data for enjoyment was collected from questionnaires by classifying free comments of the children and their mothers.

### Statistical analysis

Analyses within the intervention group were conducted by dividing this group into two categories based on the mother’s musical background. This binary variable was used as an independent variable to assess differences in exercise activity between both children and their mothers.

Baseline characteristics were reported as means and standard deviations (SD) for continuous variables and as frequencies and percentages for categorical variables. Differences between the groups at baseline were tested by Mann-Whitney U test for continuous variables and Fisher’s exact test was for categorical variables. Changes in the proportion of measurement time in SB, SS, LPA, and MVPA were modified into dichotomy variables (having a positive or negative change), and binary logistic regression was used to analyze the primary outcomes. Further, the model was adjusted for baseline level of these behaviors. After fitting logistic regression models, predicted probabilities for positive change were calculated by converting odds. Based on values of skewness and kurtosis, Wilcoxon signed-rank test was used to assess for significant differences in the changes of exercise activity (completeness) within groups and Mann-Whitney U test was used to assess differences between the groups. Fisher’s exact test was used to assess differences between the groups in fidelity. Enjoyment was described as percentage of children and their mothers in different categories based on classified comments of children and their mothers.

Two-tailed significance level of 0.05 was used for analyses. All analyses were performed using IBM SPSS Statistics 24.0.

## Results

Based on questionnaire, 25 mothers had musical activities as their job (n = 0), were studying music professionally (n = 2), or had music as a hobby (n = 23) indicating mothers’ musical background. The most common instruments among participants were piano (17 mothers, mean number of playing years was 17.6), singing lessons, choir, or band (16 mothers, 5.9 years), flute (2 mothers, 10.0 years), and violin (2 mothers, 4.5 years). Dancing (including for example ballet, couple dancing, and folk dances) was reported by 8 mothers (8.1 years) and having other movement-to-music activities (such as aerobics or dance-exergames) by 17 mothers (8.2 years). All the mothers reported listening to music regularly, mostly on the radio (68 mothers), CDs (49 mothers), streaming services (46 mothers), or live (35 mothers).

At baseline, there were no statistically significant differences between the groups of mothers or children (Table 1).

**Table 1:** Background characteristics.

	Having a musical background (n = 25)	Not having a musical background (n = 46)	p-value
Mothers			
Age (in 2015)	38.2 ± 5.4	36.1 ± 4.0	0.069^1^
Married or cohabiting	95.8 (n = 24)	93.5	1.00^2^
Number of children	2.7 ± 1.3	2.5 ± 0.8	0.76^1^
Employment, at work	70.8 (n = 24)	80.4	0.38^2^
BMI	27.3 ± 4.5	26.6 ± 5.6	0.35^1^
Children			
Age	6.7 ± 0.4	6.5 ± 0.5	0.18^1^
Gender, girls	60.0	41.3	0.15^2^
BMI-for-age	22.7 ± 4.6 (n = 13)	21.2 ± 3.1 (n = 26)	0.46^1^
Daycare or preschool	68.0	77.3 (n = 44)	0.41^2^

Data are presented mean ± SD or %.

^1^ Mann-Whitney U test

^2^ Fisher’s exact test

### Objective measurements

#### Children

At baseline week and at the first intervention week, in total 21 children in the MB group and 40 children in the NMB group had a valid accelerometer measurement. The corresponding numbers of children at the last intervention week were 14 and 32, respectively. In total, 12 children in the MB group and 29 children in the NMB group had valid accelerometer measurement in all three weeks. At baseline there were no statistically significant differences between the MB and NMB groups.

**Fig 1:**
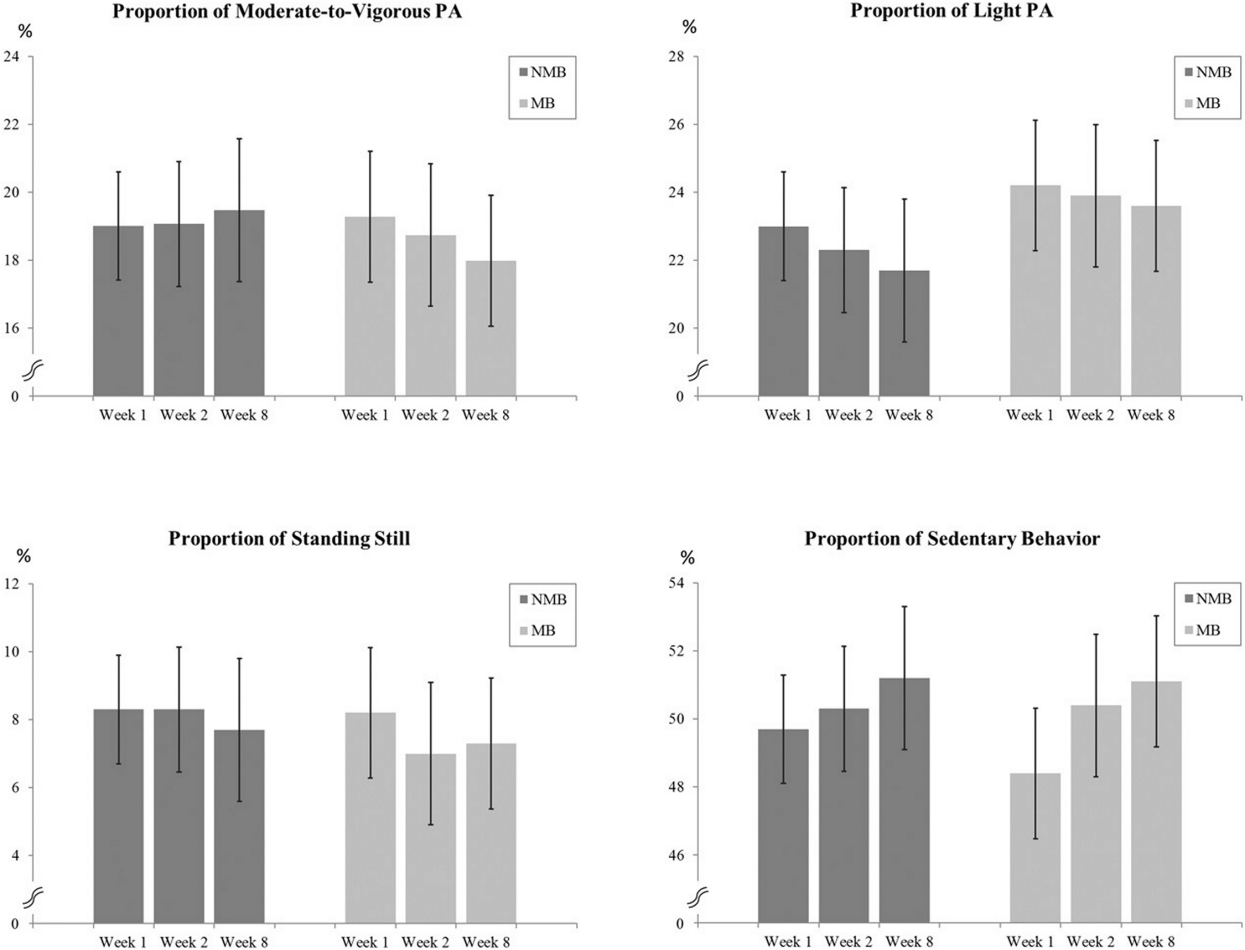
The average proportion of MVPA, LPA, SS, and SB with 95% confidence intervals (CIs) in the week 1, 2, and 8 in children who had a valid accelerometer measurement in all three weeks.

Based on the objective measurements, the average proportion of MVPA, LPA, SS, and SB with 95% confidence intervals (CIs) of each value at baseline, the first and the last intervention week are presented in Fig 1.

Regarding children, quantitative results are presented in Table 2. Statistically significant differences were found between the weeks 1 and 8 in LPA (OR = 7.64, *p* = 0.014) and between the weeks 1 and 2 in SS (OR = 0.18, *p* = 0.043). Thus, children in the MB group had greater probability to belong to category of positive change in LPA and smaller probability to belong to category of positive change in SS compared to the NMB group.

**Table 2:** Odds ratios (ORs) with 95% confidence intervals (CIs) from logistic regression models in children. Association between groups and changes in MVPA, LPA, Total-PA, SS, and SB.

		Having musical background	Not having musical background (ref. group)		
	n	positive change, n (%)	positive change, n (%)	OR (95% CI)	p-value
MVPA					
Weeks 1–2	61	11 (52)	19 (48)	1.17 (0.40–3.42)	0.77
	41	6 (51)	14 (48)	1.09 (0.28–4.19)	0.91
Weeks 2–8	41	6 (51)	14 (48)	1.06 (0.27–4.09)	0.94
Weeks 1–8	41	3 (25)	16 (55)	0.27 (0.06–1.22)	0.089
LPA					
Weeks 1–2	61	9 (43)	14 (35)	1.40 (0.47–4.13)	0.54
	41	5 (42)	8 (28)	2.07 (0.47–8.77)	0.33
Weeks 2–8	41	6 (50)	13 (45)	1.35 (0.34–5.37)	0.67
Weeks 1–8	41	7 (58)	6 (21)	7.64 (1.51–38.65)	**0.014**
Total-PA					
Weeks 1–2	61	11 (52)	26 (65)	0.60 (0.20–1.75)	0.35
	41	7 (58)	20 (69)	0.64 (0.16–2.58)	0.64
Weeks 2–8	41	6 (50)	15 (52)	1.03 (0.26–4.06)	0.97
Weeks 1–8	41	5 (42)	14 (48)	0.73 (0.19–2.88)	0.65
SS					
Weeks 1–2	61	6 (29)	21 (53)	0.27 (0.08–0.95)	**0.041**
	41	3 (25)	17 (59)	0.18 (0.04–0.95)	**0.043**
Weeks 2–8	41	8 (67)	12 (41)	2.89 (0.69–12.14)	0.15
Weeks 1–8	41	2 (17)	11 (38)	0.22 (0.03–1.47)	0.12
SB					
Weeks 1–2	61	12 (57)	24 (60)	0.93 (0.31–2.73)	0.89
	41	9 (75)	18 (62)	1.87 (0.41–8.50)	0.42
Weeks 2–8	41	6 (50)	17 (59)	0.65 (0.17–2.59)	0.55
Weeks 1–8	41	8 (66)	18 (62)	1.17 (0.28–4.87)	0.83

Models were adjusted for baseline level of the particular outcome.

Further, probability for increased MVPA was 25% in the MB group and 55% in the NMB group. The corresponding values for increased LPA was 62% and 18%, increased SS 11% and 36%, and increased SB 66% and 62%, respectively.

#### Mothers

At baseline week and at the first intervention week, in total 23 mothers in the MB group and 42 mothers in the NMB group had valid accelerometer measurement. The corresponding numbers of mothers during the last intervention week were 19 and 33, respectively. In total, 17 mothers in the MB group and 32 mothers in the NMB group had valid accelerometer measurement in all three weeks. At baseline there were no statistically significant differences between the MB and NMB groups.

**Fig 2:**
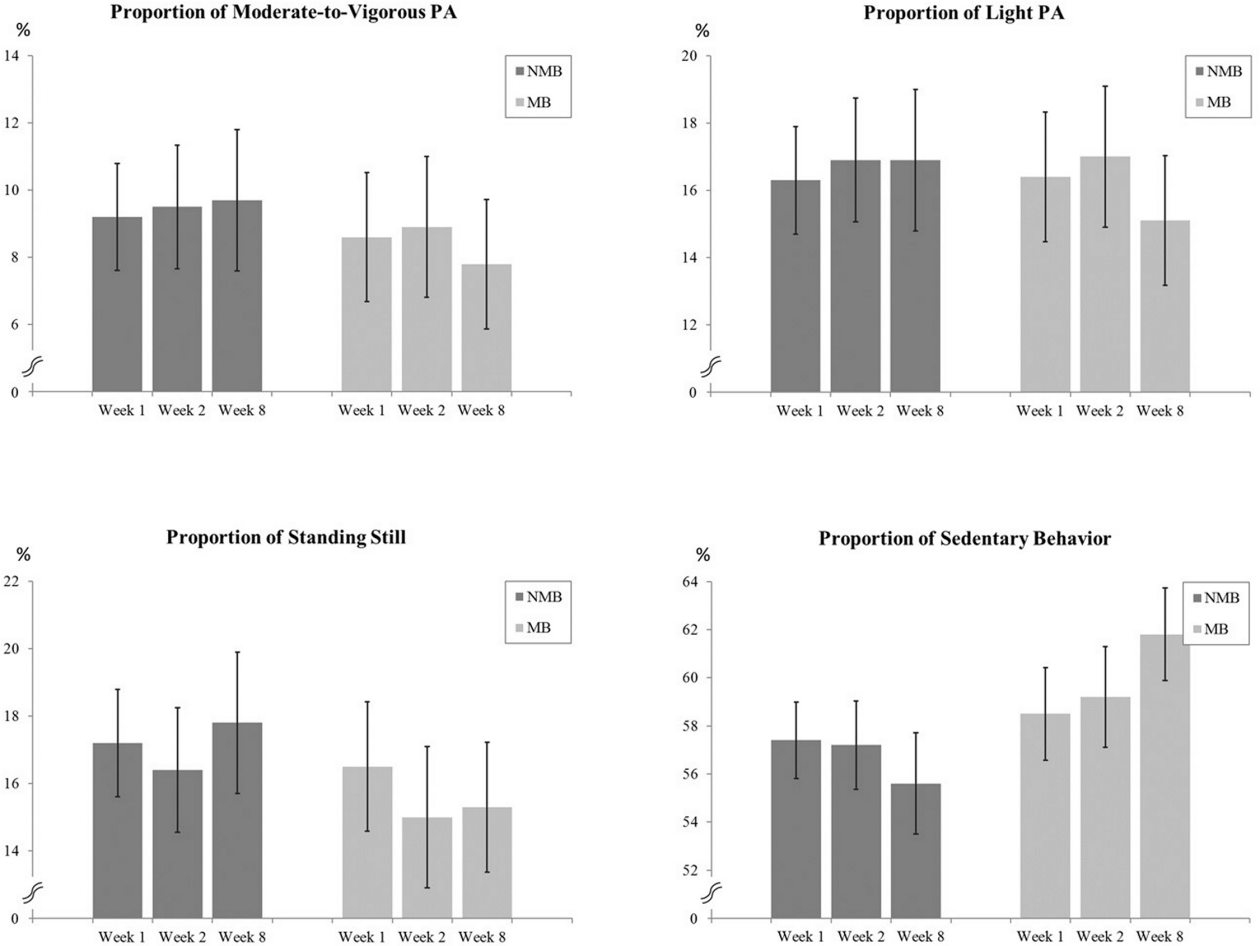
The average proportion of MVPA, LPA, SS, and SB with 95% confidence intervals (CIs) in the week 1, 2, and 8 in mothers who had a valid accelerometer measurement in all three weeks.

Based on the objective measurements, the average proportion of MVPA, LPA, SS, and SB with 95% confidence intervals (CIs) of each value at baseline, the first and the last intervention week are presented in Fig 2.

Regarding mothers, quantitative results are presented in Table 3. Statistically significant differences were found between the weeks 1 and 8 in LPA (OR = 0.21, *p* = 0.021) and in total-PA (OR = 0.13, *p* = 0.004) indicating that mothers in the MB group had smaller probability to belong to category of positive change in LPA and total-PA compared to the NMB group. We also found that between the weeks 2 and 8 mothers in the MB group had greater probability to belong to category of positive change in total-PA (OR = 3.87, *p* = 0.047) and smaller probability to belong to category of positive change in SS (OP = 0.16, *p* = 0.009) compared to the NMB group. Mothers in the MB group had greater probability to belong to category of positive change (which means negative result, i.e., growing) in SB compared to the NMB group between the weeks 2 and 8 (OR = 3.82, *p* = 0.038).

**Table 3:** Odds ratios (ORs) with 95% confidence intervals (CIs) from logistic regression models in mothers. Associations between groups and changes in MVPA, LPA, Total-PA, SS, and SB.

		Having musical background	Not having musical background (ref. group)		
	n	positive change, n (%)	positive change, n (%)	OR (95% CI)	p-value
MVPA					
Weeks 1–2	65	11 (48)	23 (55)	0.71 (0.25–2.04)	0.53
	49	8 (47)	19 (59)	0.54 (0.16–1.84)	0.32
Weeks 2–8	49	5 (29)	16 (50)	0.43 (0.12–1.52)	0.19
Weeks 1–8	49	5 (29)	17 (53)	0.32 (0.09–1.17)	0.085
LPA					
Weeks 1–2	65	12 (52)	22 (52)	0.99 (0.36–2.75)	0.98
	49	10 (59)	18 (56)	1.09 (0.32–3.69)	0.89
Weeks 2–8	49	5 (29)	17 (53)	0.35 (0.10–1.30)	0.12
Weeks 1–8	49	4 (24)	19 (59)	0.21 (0.06–0.79)	**0.021**
Total-PA					
Weeks 1–2	65	11 (48)	18 (43)	1.22 (0.44–3.39)	0.70
	49	8 (47)	12 (38)	1.46 (0.44–4.84)	0.54
Weeks 2–8	49	13 (77)	15 (47)	3.87 (1.02–14.78)	**0.047**
Weeks 1–8	49	4 (24)	22 (69)	0.13 (0.03–0.52)	**0.004**
SS					
Weeks 1–2	65	5 (22)	17 (41)	0.41 (0.13–1.31)	0.13
	49	4 (24)	11 (34)	0.60 (0.16–2.28)	0.45
Weeks 2–8	49	7 (41)	25 (78)	0.16 (0.04–0.64)	**0.009**
Weeks 1–8	49	6 (35)	17 (53)	0.40 (0.11–1.50)	0.17
SB					
Weeks 1–2	65	16 (70)	21 (50)	2.29 (0.78–6.75)	0.13
	49	12 (71)	15 (47)	2.76 (0.78–9.71)	0.11
Weeks 2–8	49	11 (65)	11 (34)	3.82 (1.08–13.55)	**0.038**
Weeks 1–8	49	12 (71)	14 (44)	3.27 (0.91–11.69)	0.069

Models were adjusted for baseline level of the particular outcome.

Further, probability for increased MVPA was 27% in the MB group and 54% in the NMB group. The corresponding values for increased LPA was 24% and 60%, increased SS 31% and 53%, and increased SB 71% and 43%, respectively.

### Exercise adherence

#### Completeness (Exercise activity)

Exercise diaries of the baseline and the first intervention weeks were returned by 24 mothers and 24 children in the MB (having a musical background) group. The corresponding values were 45 and 44 in the NMB (not having a musical background) group. Based on diaries, 15 mothers and 16 children in the MB group completed exercises at least once during the first week. Corresponding values in the NMB group were 36 mothers and 34 children. The groups did not differ in the number of completed exercises with movement-to-music video program (mothers’ *p* = 0.30, children *p* = 0.42) during the first intervention week.

Diaries from the last week were returned by 18 mothers and 17 children in the MB group and by 35 mothers and 35 children in the NMB group. Only 2 mothers and 2 children in the MB group and 5 mothers and 6 children in the NMB group reported at least one completed exercise during the last week. Exercise activity (the number of completed exercises) with movement-to-music video program was significantly lower during the last than the first intervention week in all groups which indicates poor completeness of the program. Table 4 presents changes in self-reported exercise activity (number of exercises per week) with movement-to-music video program.

**Table 4:** Changes in the number of exercises per week with movement-to-music video program.

	Having at least one completed exercise		An average number and SD of exercises		Mean (SD) and Median (min., max.) of change	*p* value^1^	*p* value^2^
	The first week	The last week	The first week	The last week			
Mothers							
Having a musical background	63%	11%	1.5 (1.4)	0.2 (0.5)	-1.8 (1.2), -2.0 (-3, 0)	0.001	0.86
Not having a musical background	80%	14%	1.9 (1.4)	0.3 (0.8)	-1.8 (1.3), -2.0 (-4, 0)	<0.001	
Children							
Having a musical background	67%	12%	1.7 (1.5)	0.1 (0.3)	-1.9 (1.4), -2.5 (-4, 0)	0.002	0.72
Not having a musical background	77%	17%	2.0 (1.5)	0.3 (0.8)	-1.8 (1.4), -2.0 (-4, 0)	<0.001	

^1^ Wilcoxon signed-rank test (changes within group)

^2^ Mann-Whitney test (changes between groups)

#### Fidelity

Questionnaires were returned by 24 children and 24 mothers in the MB group after the first intervention week and by 12 children and 14 mothers after the last intervention week. Questionnaires were returned by 44 children and 44 mothers in the NMB group after the first intervention week and by 23 children and 22 mothers after the last intervention week. Based on the questionnaires 75% of the children and 71% of the mothers in the MB group moved as instructed during the video watching during the first week. The corresponding values were 89% and 93% in the NMB group. During the last week, the corresponding values were 58% of the children and 71% of the mothers in the MB group and 96% of the children and 77% of the mothers in the NMB group. Most of those children who did not move as instructed were reported to come up with their own moves in the MB group (13% in the first and 42% in the last week). The corresponding values were 7% in the first and 0% in the last week in the NMB group. Regardless of the group only few children were reported to sitting on a chair or standing still on a floor while watching the movement-to-music video.

Based on the mothers’ assessments, most of the exercises in movement-to-music video required balance and movement control rather than endurance or strength. Compared with the children for who belonged to the NMB group (n = 43), children in the MB group (n = 24) were slightly less likely to consider balance and movement control as required feature (79% vs. 95%, *p* = 0.088) after the first intervention week. Corresponding values after the last intervention week were 50% (n = 14) vs. 77% (n = 26), *p* = 0.16. Further, most of the exercises were assessed brisk and breeze rather than leisurely and calm or intense and strenuous. Children in the MB group were less likely to consider exercises brisk and breeze compared to the children in the NMB group (after the first intervention week 67% vs. 70%, *p* = 0.79 and after the last intervention week 57% vs. 69%, *p* = 0.50).

#### Enjoyment

Mothers assessed the motivational effects of songs in the video using BMRI-2 [29,30]. Mothers who did not have a musical background were more likely to belong in the highly motivated by music group compared to those who had different kind of music-based hobbies (29% vs. 18%). Mothers who had a musical background were more likely to belong in the moderately motivated by music group compared to those who had not musical background (54% vs. 41%). The percentage of mothers who thought that motivational effects of songs were neutral was similar in both groups (29% in the MB group vs. 27% in the NMB group). Regardless of having or not having a musical background, mothers who were highly motivated by music had smaller probability to belong to category of positive change (which means negative result, i.e., growing) in SB compared to the mothers who thought that motivational effect of songs was neutral (n = 24, OR = 0.082, 95% CI 0.01–0.64, *p* = 0.017). Any other differences between motivational groups were not found.

We also asked mothers to write down children’s own comments to assess enjoyment. After the first intervention week 18 out of 25 children’s opinions were found in the MB group, corresponding number in the NMB group was 34 out of 46. After the last intervention week mothers reported 15 children’s opinions in the MB group and 20 in the NMB group. Around 30% of the children in the MB group and 19% in the NMB group considered the video childish when comments after both the first and the last weeks were taken to account. Further, 18% of the children in the MB group and 26% in the NMB group did not like the video, or said it was irritating, boring, or wearisome. However, the same amount of the children in both groups considered the video nice and easy, funny, and/or good. Regardless of the group 14% of the children liked the songs and 10% of the children liked to move and dance with the video. Nevertheless, after fitting logistic regression model (in supplementary analysis), we found that probability that total-PA increased was 50% among those who liked the video and 54% among those who did not.

After the first intervention week 16 mothers in the MB group reported their opinions in the free field of questionnaire, corresponding number of mothers in the NMB group was 23. After the last intervention week 14 mothers in the MB group and 18 in the NMB group reported their opinion. Regardless of the group 22% of mothers reported that the video was aimed at younger children, and further, if there were 3–4 years old children in the family, younger child liked the video more than the child who participated to the study. Based on mothers’ opinions, the same movements were repeated too many times and mothers assessed the video too simple for eight weeks (11%). Specifically, in the NMB group 14% of the mothers reported that the video did not inspire them to move. However, around 10% of mothers (regardless of the group) reported that the video was OK, and in addition, 9% reported that the video was good, nice, and/or easy. After fitting logistic regression model (in supplementary analysis), we found that probability that total-PA increased was 60% among those who liked the video and 41% among those who did not.

## Discussion

The aim of the present study was to investigate the effects of the movement-to-music video program on objectively measured SB, SS, LPA, and MVPA of the children and their mothers according to mothers’ musical background. The present study also reported completeness, fidelity, and enjoyment of the movement-to-music video program.

Overall, as a summary of the major findings those children whose mothers had musical background were more likely to increase their LPA, but not MVPA compared to children whose mothers did not have musical background. In addition, mothers in the MB group were more likely to decrease their MVPA and LPA compared to the mothers in the NMB group. Both children and their mothers in the MB group had greater probability to increase their SB compared to the NMB group. The secondary outcomes were in line with major findings: exercise activity (completeness) and fidelity were lower among the MB group compared to the NMB group. Further, mothers in the NMB group were more likely to belong to highly-motivated-by-music group and one-fourth of children in the NMB group were reported to like the video. The main problems with the video and exercise adherence seemed to be related with the children’s age (video was targeted to younger children) and exercises which were felt to repeat themselves.

We suggest that if the video would have been more interesting and more engaging for older children, the possibility of having a higher participation rate until the end would have been greater. However, video was carefully pretested both with professional physiotherapists [25] and mother-child pairs [31].

Mothers who did not have music-based hobbies seemed to be more physically active and have less sedentary time compared to those who had music-based hobbies. We suggest that large part of the music-based hobbies, specifically, listening, singing and playing an instrument, are quite sedentary in nature. However, the case was not as clear with the children and we do not know whether the children themselves had music-based hobbies. Parents act as a role model for their children both in PA and SB [32–35], and they have an important role in their child’s musical performance [36] as well.

When discussing movement-to-music activities or dancing, Temmerman (2000) found that a lack of space to move freely and aspects of how moving experiences are organized and managed are related to the least-enjoyed aspects of music-based activities [17]. Our results established that engaging in moving exercises which children and their mothers perceived lack of variety decreased over time. However, although one-fifth of the children did not like the video or said it was boring, dislike was more related to the current video and guided exercises rather than moving with music as such. This is also in line with Temmerman (2000) who found that children tend to have positive attitudes towards musical activities which provide them to move freely instead of guided exercises [17].

As a contrast for earlier studies, those children who nominated that the video was nice and easy, funny, and good, had smaller probability to increase their total-PA compared to those who did not like the video. This result is opposite to Remmers et al. (2015) who found that enjoyment of PA was related with active behavior [5]. This, however, may indicate that children’s daily PA in all intensity levels moved them more than our video program at the end of the intervention. Among mothers those who liked the video had higher probability to increase their total-PA than those who did not like it, and this result is similar than what was found in earlier studies [13,23].

### Limitations

The movement-to-music video program used in the present study was somewhat repetitive in nature. Since we know how important role motivation plays for decreasing SB and increasing PA, this may have influenced to children’s and their mothers’ exercise motivation and adherence. Among adults Heisz et al. (2016) concluded that changes in workload predicted changes in exercise enjoyment in sedentary adults [37]. Further, Gao, Podlog, & Huang (2013) found that intrinsic motivation was the significant predictor for both PA enjoyment and MVPA in children [38]. Gao, Zhang, & Stodden (2013) also found that children had longer MVPA time during aerobic dance session compared to interactive dance game, but their enjoyment was higher during dance game [39]. Even if children and their mothers in our study were able to choose suitable movements for themselves from one to three variations, it seemed not to be enough to increase workload or exerts during the intervention. However, we suggest that individuals with musical background might need stronger musical experiences or growing challenges (such as interactive playing [39]) to motivate and decrease their SB and increase PA. However, to our knowledge, differences in exercise adherence between individuals with or without musical background has not been studied previously and thus this study provides valuable information to be utilized in further studies.

## Conclusion

To our knowledge, this is the first study comparing SB and PA among mother-child pairs according to mothers’ musical background. As a conclusion, the present results showed that children and mothers without musical background were more interested in movement-to-music exercises. In further studies it would be important to evaluate an effect of children’s own music-based hobbies on their SB and PA. In addition, it would be reasonable to adjust the exercises and music more challenging according to the musical background of the participants.

## Abbreviations

BMI: body mass index
BMRI: Brunel music rating inventory
CI: confidence interval
LPA: light physical activity
MAD: mean signal amplitude deviation
MB: musical background group
MET: metabolic equivalent
MVPA: moderate to vigorous physical activity
NMB: not musical background group
PA: physical activity
RCT: randomized controlled trial
SB: sedentary behavior
SD: standard deviation
SS: standing
Total-PA: light, moderate, and vigorous physical activity
